# Direct Conjugation of Resveratrol on Hydrophilic Gold Nanoparticles: Structural and Cytotoxic Studies for Biomedical Applications

**DOI:** 10.3390/nano10101898

**Published:** 2020-09-23

**Authors:** Iole Venditti, Giovanna Iucci, Ilaria Fratoddi, Manuela Cipolletti, Emiliano Montalesi, Maria Marino, Valeria Secchi, Chiara Battocchio

**Affiliations:** 1 Department of Sciences, Roma Tre University of Rome, 00146 Rome, Italy; giovanna.iucci@uniroma3.it (G.I.); manuela.cipolletti@uniroma3.it (M.C.); emiliano.montalesi@uniroma3.it (E.M.); maria.marino@uniroma3.it (M.M.); valeria.secchi@uniroma3.it (V.S.); chiara.battocchio@uniroma3.it (C.B.); 2 Department of Chemistry, Sapienza University of Rome, 00185 Rome, Italy; ilaria.fratoddi@uniroma1.it

**Keywords:** gold nanoparticles, resveratrol, conjugation, immobilization, bioconjugates, drug delivery systems

## Abstract

Strongly hydrophilic gold nanoparticles (AuNPs), functionalized with citrate and L-cysteine, were synthetized and used as Resveratrol (RSV) vehicle to improve its bioavailability. Two different conjugation procedures were investigated: the first by adding RSV during AuNPs synthesis (1) and the second by adding RSV after AuNPs synthesis (2). The two different conjugated systems, namely AuNPs@RSV1 and AuNPs@RSV2 respectively, showed good loading efficiency (η%): η_1_ = 80 ± 5% for AuNPs@RSV1 and η_2_ = 20 ± 3% for AuNPs@RSV2. Both conjugated systems were investigated by means of Dynamic Light Scattering (DLS), confirming hydrophilic behavior and nanodimension (<2R_H_> _1_ = 45 ± 12 nm and <2R_H_> _2_ = 170 ± 30 nm). Fourier Transform Infrared Spectroscopy (FT-IR), Synchrotron Radiation induced X-Ray Photoelectron Spectroscopy (SR-XPS) and Near Edge X-ray Absorption Fine Structure (NEXAFS) techniques were applied to deeply understand the hooking mode of RSV on AuNPs surface in the two differently conjugated systems. Moreover, the biocompatibility of AuNPs and AuNPs@RSV1 was evaluated in the concentration range 1.0–45.5 µg/mL by assessing their effect on breast cancer cell vitality. The obtained data confirmed that, at the concentration used, AuNPs do not induce cell death, whereas AuNPs@RSV1 maintains the same anticancer effects as the unconjugated RSV.

## 1. Introduction

Gold nanoparticles (AuNPs) have amazing chemical–physical features that make them appealing for biotechnology and medical applications [1,2,3,4,5]. The advanced state of their synthetic chemistry allows strict control on shape, dimension and surface functionalization using, among others, thiols or organic molecules as capping agents that could be chemically modified to link targeting biomolecules or/and drugs [6,7,8,9].

The opportunity to conjugate molecules on nanosupports through stable interactions (e.g., electrostatic or covalent interactions) is useful for applications such as drug delivery systems (DDSs) allowing stable molecule/carrier conjugates and facilitating the bioavailability and targeted delivery of molecules [10,11,12]. Although there are many issues that need to be addressed, such as the biological interaction of the nanocarrier in patients, the knowledge of the molecular mechanisms that drive nanoparticle-cell interactions, the barriers to marketing related to manufacturing, costs and regulatory standards, today research is investing heavily in this area, particularly seeking to minimize the complexity of nanomaterials by taking into account the final form and dosage for human use, so that a formulation has the potential to be translated into clinically applicable therapies [13,14]. The development of an effective method for the enhancement of therapeutic agents delivery was deeply investigated, then interesting complex platforms were proposed in the literature such as, for example, functionalized AuNPs, composites between AuNPs and copolymeric micelles, core–shell systems and photonic and plasmonic hybrid materials [15,16,17,18]. The success of AuNPs largely depends on their degree of inherent toxicity and studies on the toxicological profile are discussed preceding their usage in cancer management [19]. It has been proven that the cytotoxicity of AuNPs is closely related to nanoparticle size, surface charge, and functional groups [20,21]. Moreover, the surface functionalization of gold nanoparticles is crucial for the effective utilization of these materials in health-related applications and hydrophilic molecules, such as thiols, amines or polymers, can provide a suitable way to introduce reactive functional groups that can be utilized for targeting (e.g., antibodies, peptides, aptamers) and conjugating therapeutic agents (e.g., drugs, radionuclides, photosensitizers) [22,23,24,25].

The plant-derived polyphenols, mostly found in edible vegetables and fruits, are widely reported as potential anticancer molecules [26,27]. Among others, resveratrol (*trans*-3,5,4′-trihydroxystilbene; RSV) deserves particular attention for the plethora of metabolic effects with potential health benefits reported in literature, which include the prevention of initiation, promotion, and progression of breast cancer [28,29,30]. Several action mechanisms have been suggested to be at the root of RSV anticancer effects, including the ability of high RSV concentration (i.e., >10 µM) to reduce breast cancer cell viability [31]. Nevertheless, no clinical applications to cancer treatment have originated from this knowledge yes, probably due to the extensive metabolism which RSV undergoes in the human gut and liver after absorption, which strongly reduces its bioavailability [29,32,33,34]. The problem could be solved by exploiting a highly hydrophilic vehicle, such as AuNPs or liposomes, to deliver a poorly bioavailable compound, as reported for other drugs [35,36,37,38,39,40,41].

As reported by Thipe and collaborators [42], the combination of RSV with gold metal is an ancient medical protocol extensively used in the Indian Ayurvedic medicine, referred to as Swarna Bhasma, to treat various diseases and disorders. Nonetheless, very few scientific publications (i.e., less than 20 present in the pubmed site) addressed the efficacy of gold-conjugated RSV nanoparticles as a therapeutic agent. Most of these papers focused on the ability of RSV to maintain its antibacterial activity [43], antioxidant properties [44,45], anti-hepatoma [46], anti-breast cancer cell invasion [47], and anti-diabetic [48] effects.

In this work, AuNPs functionalized with hydrophilic capping agents, i.e., sodium citrate (cit) and L-Cysteine (L-cys), were synthesized and deeply investigated. These AuNPs were used to conjugate RSV, improving its bioavailability. Two different procedures were studied to optimize the RSV loading efficiency (η%) on AuNPs: the first conjugation procedure by adding RSV in reaction mixture during the AuNPs synthesis and the second by adding RSV in AuNPs water suspension after their synthesis and purification. In this way two different conjugated systems, AuNPs@RSV1 and AuNPs@RSV2, were prepared. The best loading was obtained by AuNPs@RSV1. Deeply structural characterization of AuNPs@RSV1 and AuNPs@RSV2 was performed to confirm the RSV stability and to investigate its interaction with gold surface in the two systems. Moreover, the biocompatibility of AuNPs and AuNPs@RSV1 was evaluated in a concentration range of 1.0–45.5 µg/mL by assessing their effects on breast cancer cell, MCF-7, viability.

## 2. Experimental Part

### 2.1. Materials and Methods

Sodium citrate (cit) (Na_3_C_6_H_5_O_7_), L-Cysteine (L-cys) (C_3_H_7_NO_2_S), tetrachloroauric(III) acid trihydrate (HAuCl_4_·3H_2_O), sodium borohydride (NaBH_4_), and Resveratrol (RSV) have been used as received (Merck reagent grade).

The Bradford protein assay was purchased from Bio-Rad Laboratories (Hercules, CA, USA), as well as the chemiluminescence reagents for Western blot Clarity Western ECL Substrate. The Anti-α-tubulin antibody was purchased from Merck (Darmstadt, D) (cat.# T9026, clone DM1A), while the poly [ADP-ribose] polymerase 1 (PARP-1) polyclonal antibody was obtained from Cell Signalling Technologies (Leiden, NL, The Netherlands) (cat.#9542). All the other products were from Sigma-Aldrich (St. Louis, MO, USA). Analytical and reagent grade products were used without further purification.

UV-vis spectra were run in H_2_O solution by using quartz cells with a Shimadzu 2401 PC UV-vis spectrophotometer. Fourier Transform Infrared Spectroscopy (FT-IR) spectra have been recorded as films deposited by casting from water suspension, with a Bruker Vector 22 equipped with a DTGS detector and with a Specac P/N 19650 series monolayer/grazing angle accessory. The size distribution of AuNPs in H_2_O solution has been investigated by means of the Dynamic Light Scattering (DLS) technique by using a Brookhaven instrument (Brookhaven, NY, USA) equipped with a 10 mW HeNe laser at a 632.8 nm wavelength and at a temperature of (25.0 ± 0.2) °C. Correlation data have been acquired and fitted in analogy to our previous work [49].

Synchrotron Radiation-induced X-ray Photoelectron Spectroscopy (SR-XPS) measurements were carried out at the PM4-LowDosePES beamline at Helmholtz-Zentrum Berlin (BessyII Synchrotron Radiation facility), allowing for a lower flux on the sample, mandatory to avoid damaging biomolecules conjugated to AuNPs. This soft X-ray bending magnet beamline is equipped with a Plane Grating Monochromator operating in collimated light (collimated PGM). The LowDose PES end-station is equipped with a SES100 hemispherical analyzer, whose Energy Resolution was estimated as 0.2 eV. A photon energy of 586 eV was used for C1s, S2p, Au4f, N1s and O1s spectral regions with energy resolution NE = 0.22 eV. Calibration of the energy scale was made referencing all the spectra to the gold Fermi edge of an Au reference sample, and the Au4f_7/2_ signal related to metallic gold was always found at 83.9 eV. Curve-fitting analysis of the C1s, O1s, S2p, Au4f, N1s spectra was performed using Gaussian curves as fitting functions. The S2p_3/2_,_1/2_ doublet was fitted by using the same full width at half maximum (FWHM) for both components, a spin–orbit splitting of 1.2 eV, and a branching ratio (S2p_3/2_/S2p_1/2_) of 2. For Au4f_7/2_,_5/2_ doublet, a splitting of 3.6 eV, a branching ratio Au4f_7/2_/Au4f_5/2_ of 4/3 and the same FWHM values for both spin–orbit components were used. When several different species were identified in a spectrum, the same FWHM value was used for all individual photoemission bands.

Near Edge X-ray Absorption Fine Structure (NEXAFS) spectroscopy experiments were performed at the ELETTRA storage ring at the BEAR (bending magnet for emission absorption and reflectivity) beamline, installed at the left exit of the 8.1 bending magnet exit. The apparatus is based on a bending magnet as a source, a beamline optics delivering photons from 5 eV up to about 1600 eV with selectable degree of ellipticity. The carbon K-edge spectra were collected at normal (90°), magic (54.7°) and grazing (20°) incidence angles of the linearly polarized photon beam with respect to the sample surface. The photon energy and resolution were calibrated and experimentally tested at the K absorption edges of Ar, N_2_ and Ne. The spectra were then normalized subtracting a straight line that fits the part of the spectrum below the edge and assessing to 1 the value at 330.00 eV.

### 2.2. Synthesis and Purification of AuNPs

The AuNPs stabilized with cit and L-cys have been prepared and characterized in analogy to literature reports [50,51]. Briefly: 1.47 g of cit (0.01 M) was dissolved in 50 mL of distilled water, 0.006 g of L-cys (0.002 M) in 25 mL and finally 0.42 g of tetrachloroauric acid (0.05 M) in 25 mL of water. 25 mL of L-cys solution, 10 mL of cit solution and 2.5 mL of tetrachloroauric acid solutions were added sequentially in a 100 mL flask, provided with a magnetic stir. The solution was degassed with Argon for 10 min, then 4 mL of sodium borohydride solution (0.0030 g in 4 mL, 0.00008 mol) were added. The mixture was allowed to react at room temperature for 2 h and at the end the brown solid was recollected and purified by centrifugation (13,000 rpm, 10 min, 4 times with deionized water). AuNPs: UV-vis (λ_max_ [nm], H_2_O): 550 nm.

### 2.3. AuNPs@RSV Preparations and Characterizations

Two different procedures were investigated to optimize the RSV loading. (1) AuNPs@RSV1: conjugation by chemical adsorption during AuNPs synthesis; AuNPs synthesis was carried out following the same procedures but including RSV water solution (1 mL 0.02 M) in the reagent mixture. All tests were performed in triplicate [52]. (2) AuNPs@RSV2: conjugation by physical adsorption on AuNPs post their synthesis: AuNPs and RSV (AuNPs/RSV = 1/2 *w*/*w*) were mixed and stirred at room temperature for 4 h. At the end of the contact time, the solutions were centrifuged for 30 min (20 °C, 13,000 rpm). All tests were performed in triplicate. The loading and the loading efficiency (η) were calculated by using calibration curves, as reported in the (Supporting Information Figure S1) and calculated in reference to our previous works [40,52]. For each sample, three independent measurements were carried out and the mean value and standard deviation are reported.

### 2.4. Cell Lines and In Vitro Assays

#### 2.4.1. Cell Culture and Stimulation

Human breast cancer cells MCF-7 (ATTC, LGC Standards S.r.l., Milano, Italy) were grown in air containing 5% CO_2_ in modified, phenol red-free, Dulbecco’s Modified Eagle’s Medium (DMEM) medium. Ten percent (vol/vol) of charcoal-stripped fetal calf serum, L-glutamine (2 mM), gentamicin (0.1 mg/mL), and penicillin (100 U/mL) were added to the media before use. Cells were used at passage 13–17. The cell line authentication was periodically performed by amplification of multiple short tandem repeat loci by BMR genomics S.r.l (Padova, Italy). Cells were treated for 48 h with either vehicle (ethanol [EtOH]/DMEM, 1:10; vol/vol) or 17β-estradiol (E2, 10 nM) as cell growth inducer (positive control) or Staurosporine (STS, 10 or 100 nM) as cell death inducer (negative control) or RSV (0.1–10 µM) or AuNPs (1, 3, 9.1, 45.5, 91 and 910 µg/mL) or AuNPs@RSV1 (9.1, 45.5 and 91 µg/mL).

#### 2.4.2. Western Blot Assay

Briefly, after the treatments, cells were lysed and protein were solubilized in the YY buffer (50 mM HEPES at pH 7.5, 10% glycerol, 150 mM NaCl, 1% Triton X-100, 1 mM EDTA, and 1 mM EGTA) containing 0.70% (wt/vol) sodium dodecyl sulfate (SDS). Total proteins were quantified using the Bradford protein assay. Solubilized proteins (20 µg) were resolved by 7% or 15% SDS-polyacrylamide gel electrophoresis at 100 V for 1 h at 24.0 °C and then transferred to nitrocellulose with the Trans-Blot Turbo Transfer System (Bio-Rad) for 10 min. The nitrocellulose was treated with 5% (wt/vol) bovine serum albumin in 138.0 mM NaCl, 25.0 mM Tris, pH 8.0, at 24.0 °C for 1 h and then probed overnight at 4.0 °C with anti-PARP-1 (final dilution, 1:1000) antibody. Moreover, anti-α-tubulin (final dilution, 1:30,000) antibody was used to normalize the protein loaded. The antibody reaction was visualized with the chemiluminescence western blot analysis detection reagent (Amersham Biosciences, Little Chalfont, UK). The densitometric analyses were performed by the ImageJ software for Microsoft Windows (National Institute of Health, Bethesda, MD, USA).

#### 2.4.3. Propidium Iodide (PI) Assay

After being grown up to 80% confluence in 96-well plate, MCF7 cells were treated as reported above. Fixation and permeabilization were performed through ice cold EtOH 70% for 15 min at −20 °C. After EtOH solution removal, the cells were incubated with PI buffer for 30 min in the dark. Again, solution removal was performed, and the cells were rinsed with PBS solution. The fluorescence was revealed (excitation wavelength: 537 nm; emission wavelength: 621 nm) with TECAN Spark 20 M multimode microplate reader (Life Science, Italy).

#### 2.4.4. Statistical Analysis.

The statistical analyses were performed by Student’s *t*-test, to compare two sets of data, or by ANOVA followed by Bonferroni test, to compare different group of data, by INSTAT software system for Windows. In all cases, *p* < 0.05 was considered significant.

## 3. Results and Discussion

### 3.1. Synthesis and Characterizations of AuNPs and AuNPs@RSV

**Figure 1:**
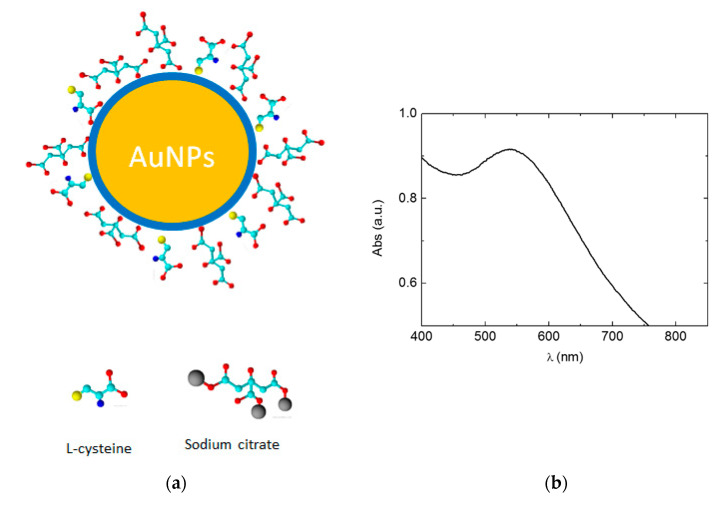
(**a**) Scheme of gold nanoparticles (AuNPs); (**b**) AuNPs Uv-vis spectrum in water with Table 550 nm.

Highly hydrophilic AuNPs were synthetized using cit and L-cys as capping agents (see Figure 1a) to assure water dispersion by cit and modulation of size by L-cys. Moreover, both of these molecules are biocompatible and are widely used for biotechnological applications, as reported in several recent papers [53,54]. UV-vis spectrum in water confirmed AuNPs nanodimension and hydrophilia, showing a typical localized surface plasmon resonance (LSPR) at 550 nm, (see Figure 1b). This result revealed that these AuNPs are particularly suitable for drug delivery, being able to act as a vehicle for biologically active but minimally hydrophilic and therefore minimally bioavailable molecules [37].

The loading protocols for AuNPs and the RSV were performed following two different approaches to compare and optimize the load: (1) direct interaction during the synthesis or (2) interaction post synthesis, giving as products the AuNPs@RSV1 and AuNPs@RSV2 respectively.

Both approaches have advantages and disadvantages. The loading of the drug carried out by incorporating RSV into the synthesis mixture allows a greater interaction with the metal particles that are gradually formed and that are immediately in contact both with RSV and with the capping agents, at the same time. The drawback could be a degradation of resveratrol under the strongly reducing conditions of the synthesis of AuNPs using sodium borohydride.

From this point of view, the post synthesis loading is safer and more conservative than the first approach. However, it has the disadvantage of putting the particles already covered by capping agents in contact with the RSV: surely, the interaction between the golden surface and the RSV is expected to be less effective. Of course, if the interaction occurred between the capping agent and RSV and not between gold nanoparticles surface and RSV, the problem would be overcome.

**Figure 2:**
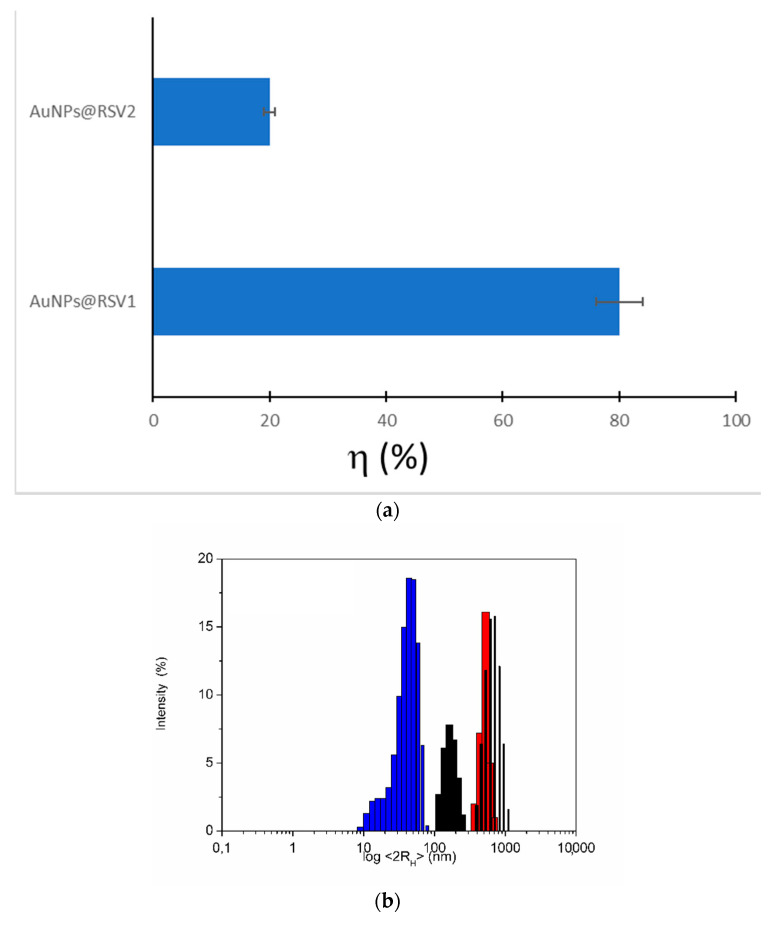
(**a**) Loading efficiency η (%) for AuNPs@RSV1 (η (%) = 80 ± 5%) and AuNPsRSV2 (η (%) = 20 ± 3%); (**b**) <2R_H_> in water of AuNPs in red (530 ± 60 nm nm), AuNPs@RSV1 in blue (45 ± 12 nm) and AuNPs@RSV2 in black (170 ± 30 nm).

For this reason, the two approaches have been developed and studied from a structural point of view using surface-sensitive techniques, that allow to probe the interface between RSV and AuNPs. Keeping the contact conditions similar, especially regarding concentrations, time and temperature, a different load efficiency was observed, strongly in favour of the contact approach during the synthesis. The value of the loading efficiency η (%), reported in Figure 2a, can be calculated as follows [52]:

η (%) = (m_loaded drug_/m_drug_) × 100

where m_loaded drug_ is the drug mass loaded on AuNPs and m_drug_ is the mass of drug used in the loading procedure. Figure 2a shows the η (%) for the two systems in comparison: AuNPs @ RSV1 produces η (%) = 80% and AuNPs @ RSV1 has η (%) = 20%.

Both systems were characterized in depth to understand the chemical interaction between AuNPs and RSV. DLS studies were performed in a water suspension and showed a dimensional increase for AuNPs@RVS1 and AuNPs@RSV2, compared with the AuNPs alone, as reported in Figure 2b. In fact, the conjugation involves a different degree of hydration of the particles and induces differences in hydrodynamic diameter (<2R_H_>), as reported in literature [49]. Moreover, the presence of the RSV during the synthesis does not affect the hydrophilic properties of the AuNPs and on the contrary induces their greater stabilization: since during the synthesis they encounter more capping agents, cit, L-cys and RSV, the dimensions are smaller and the system more monodisperse.

These first features of the two conjugated systems, which present both dimensional and RSV loading differences, highlighted the importance of deepening the study from the structural point of view of the AuNPs–RSV interaction, which is also important for the subsequent biological application.

### 3.2. Structural Characterizations of AuNPs and AuNPs@RSV

#### 3.2.1. SR-XPS Characterization

The high spectral resolution and photon flux of synchrotron radiation-induced XPS are extremely well suited to investigate the interaction at the interface between thiols and gold, allowing to probe the chemistry occurring at the headgroup–metal interface in capped nanoparticles. Moreover, the high resolution of SR-XPS measurements allows to individuate signal components related to the same chemical element belonging to different functional groups, providing essential information about the composition of complicated systems such as AuNPs@RSV1 and AuNPs@RSV2. SR-XPS experiments were carried out at the C1s, O1s, S2p, N1s and Au4f core levels on AuNPs@RSV1 and AuNPs@RSV2 deposited onto a TiO_2_/Si(111) wafer surface by following a drop-casting procedure. Due to the complex chemical composition of the functionalized nanoparticles, pristine RSV and AuNPs deposited with the same procedure onto TiO_2_/Si(111) wafer surfaces were also measured; the obtained data were used as standard for the interpretation of the signals arising by the functional NPs, as described in detail in the following. C1s, O1s, N1s, Au4f and S2p spectra were analyzed by following a peak-fitting procedure, allowing to individuate the components arising by chemical elements with different atomic environment (i.e., in different functional groups); all peak positions BE (Binding Energies), FWHM values, atomic ratios (relative intensities) and assignments are reported in Supporting Information Table S1.

**Figure 3:**
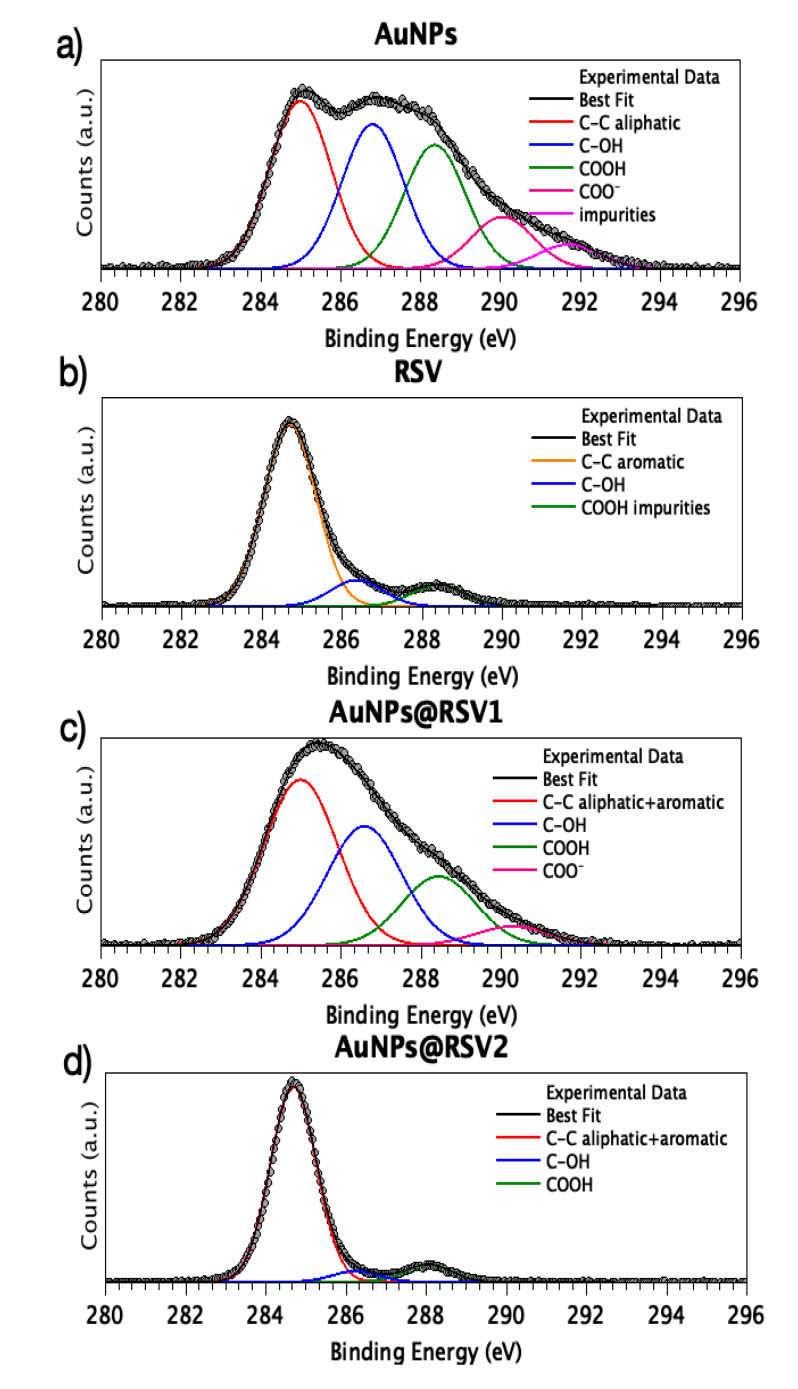
C1s spectra of (**a**) AuNPs, (**b**) Resveratrol (RSV), (**c**) AuNPs@RSV1 and (**d**) AuNPs@RSV2.

C1s spectra of AuNPs, RSV and AuNPs@RSV1 and AuNPs@RSV2 are reported in Figure 3; the AuNP spectrum (Figure 3a) appears composite and at least five spectral components can be individuated by following a peak-fitting procedure: the peak at lower BE (285.0 eV) is due to aliphatic C–C groups of citrate and impurities, always found on samples prepared in air; the peak at about 286.8 eV is attributed to C–OH functional groups of citrate and C–N groups of cysteine; the contribution at 288.4 eV is attributed to COOH end groups of cysteine and the signal at about 290 eV is associated with carboxylate groups of citrate; the last component of low intensity is due to impurities on the sample surface [55,56]. Res C1s spectrum, reported in Figure 3b), shows as expected a main peak at 284.70 eV associated with C–C aliphatic, a component around 286.40 eV due to C-OH groups, and a small contribution at higher BE values (288.4 eV) attributed to oxidized carbon-containing contaminants on the sample surface. As for the C1s spectrum of AuNPs@RSV1 (Figure 3c), four components can be individuated, and interpreted as resulting from contributions from the two previously described signals. The first peak, centered at 285.0 eV, is attributed to a mix of aliphatic and aromatic C-C arising by AuNPs (citrate C–C aliphatic) and Res (aromatic C); actually, this signal is larger (FWHM = 2.17 eV) than the C–C signals observed for AuNPs (FWHM = 1.81 eV) and Res (FWHM = 1.54 eV), as expected since aliphatic and aromatic C–C differs for 0.3 eV. The component at 286.60 eV is still assigned to C–OH groups, and, alike the C–C peak, is more intense than the same feature observed for the AuNPs and Res spectra. The third and fourth components (288.4 eV and 290.3 eV BE) are associated with carboxyl groups of the cysteine and citrate molecules, as in AuNPs, and are here less intense (due to the intensity growth of C–C and C–OH signals). Finally, the C1s spectrum of AuNPs@RSV2 (reported in Figure 3d) has almost the same shape as the C1s spectrum of pristine RSV; by applying the peak fitting procedure, three components can be individuated, with BE values analogous to those already described for RSV; noticeably, the two spectra differ for the intensity of the C–OH component, which is depressed in AuNPs@RSV2 due to the enhancement of C–C and COOH signals, due to cysteine and citrate molecules. The very small contribution of citrate and cysteine signals to the AuNPs@RSV2 C1s spectrum is due to the steric hindrance of RSV molecules, which aggregate on the AuNPs surface as an external shell; this hypothesis is in excellent agreement with the synthetic procedure consisting of the adding of RSV after the complete capping of AuNPs with cysteine and citrate. The permanence of all RSV-related signals in both AuNPs@RSV systems clearly indicates that RSV molecules interact with AuNPs with weak chemical forces, then the molecular structure and functional groups of RSV are fully conserved in the adduct formation; this observation is crucial in order to evaluate the maintaining of the resveratrol biological activity.

SR-XPS analysis of all other core level signals confirms the chemical and molecular stability of AuNPs upon interaction with RSV in both conjugated systems, as well as the absence of strong chemical interactions between RSV molecules and stabilized gold nanoparticles surface. As an example, O1s, N1s, S2p and Au4f spectra collected on AuNPs@RSV1 are reported in the Supporting Information Figure S2. To summarize, all O1s signals show two spectral components indicative of organic-like oxygen at about 532 eV (always assigned to C=O functional groups of citrate and cysteine) and at nearly 533 eV respectively, indicative of –OH groups arising by RSV [55,56,57] as reported in Supporting Information Table S1, the atomic ratio C=O/C–OH decreases, as expected, as the RSV molecules are grafted to the AuNPs (AuNPs: C=O/C–OH = 3.0/1.0, AuNPs@RSV1: C=O/C–OH = 2.3/1.0). N1s spectra of AuNPs and AuNPs@RSV1, AuNPs@RSV2 have two components both indicative of cysteine presence and stability: a main signal at about 399.2 eV due to ammine terminal groups of cysteine, and a small peak at 400.7 eV usually attributed to charged amine groups [50,56]. As for S2p spectra, the B.E. position of S2p_3/2_ signal, taken as reference for the S2p_3/2-1/2_ spin–orbit pair, indicates whether the sulfur atom is covalently bonded to the metal surface or not. For thiols chemisorbed on metals, as well as for thiols covalently bonded to metal nanoparticles surface, an S2p_3/2_ BE value of nearly 161.5 eV is expected [58]; S2p_3/2_ signals around 163 eV are usually assigned to physisorbed thiols or thiolates [58]. Both components are observed for all nanoparticles samples, suggesting that the cysteine interaction with gold atoms at NPs surface is stable and not affected by the AuNPs functionalization with Res. Au4f core level spectra are all composed of two spin–orbit doublets (Au4f_7/2_, Au4f_5/2_); the more intense Au4f_7/2_ component, due to metallic Au(0) atoms, is usually taken as reference (BE = 83.92 eV). As expected for small nanoparticles, the high surface to volume ratio allows us to observe also the Au4f signal related to substrate-thiol interface atoms [50]: the spin–orbit pairs at higher BE values (Au4f_7/2_ = 84.8 eV) clearly appear as a pronounced shoulder on all the measured spectra.

#### 3.2.2. FT-IR Characterization

**Figure 4:**
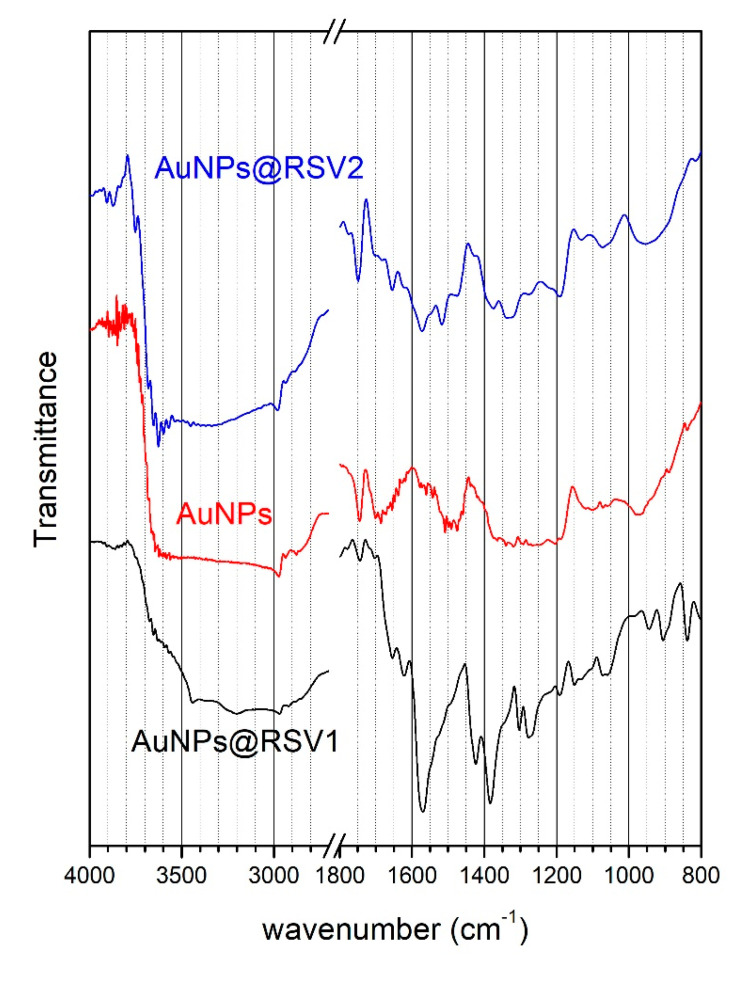
Fourier Transform Infrared Spectroscopy (FT-IR) spectra in the 2000–600 cm^−1^ range of samples AuNPs, AuNPs@RSV1 and AuNPs@RSV2. Markers represent peaks related to resveratrol.

The FT-IR spectra of samples AuNPs, AuNPs@RSV1 and AuNPs@RSV2 were recorded in order to detect the functional groups present on the AuNPs surface and hence the molecules anchored; results are shown in Figure 4.

The main peaks in the spectrum of AuNPs@RSV1 can be attributed to contributions from the cit anion. At high wavenumbers (not shown in Figure 4), the peaks located at 3450 and 3200 cm^−1^ are due to stretching vibration of the O–H bond, the peak at 2966 cm^−1^ to stretching of the C-H bond; all these peaks appear in the same position in the IR spectra of sodium citrate [59]. The intense peaks located at 1575 and 1420–1380 cm^−1^ are related to asymmetric and symmetric C=O stretching vibrations of the carboxylate anion; carboxylates are present in both cit and L-cys in its zwitterionic form. For L-cys, symmetric and asymmetric bending vibrations of the N-H bond in the ammonium cation are expected at 1600 and 1545 cm^−1^ respectively; both peaks can be detected as shoulders on the high and low energy sides of the main peak at 1575 cm^−1^. Other weak skeletal vibrations at 1280, 1270, 1194, 1150, 1070, 910, 840 cm^−1^ in the spectrum of AuNPs@Res1 correspond to similar peaks detected in the spectrum of sodium cit, which can be considered the main component of the nanoparticle surface. However, contributions from RSV to the IR spectrum of AuNPs@Res1 could also be expected. The IR spectrum of RSV is dominated by the bands related to the phenol groups. According to literature [60], C=C stretching peaks related to the aromatic rings are found at 1611 cm^−1^, 1583 cm^−1^, and 1520 cm^−1^; these peaks could be partially superimposed on the main asymmetric C=O stretching vibration of the carboxylate anion of cit. Moreover, C-O stretching and O-H bending vibrations of the phenolic hydroxyl group produce intense bands that are found at 1155 cm^−1^ and 1377 cm^−1^, respectively; the latter superimposes with the symmetric C=O stretching vibration of carboxylate. Finally, the IR spectrum of resveratrol shows peaks at 965, 831 and 670 cm^−1^; the first is typical of alkene in trans-configuration, the second of arenes conjugated to the olefinic groups and the third corresponds to =C-H of deformation bands the olefinic group. It is worth noting that weak peaks at 1155, 950, 830 and 630 cm^−1^ (marked with * in Figure 4) are present in the spectrum and could be attributed to RSV. Finally, a low intensity peak can be detected at 1740 cm^−1^, a feature that can be attributed to C=O stretching vibration of the protonated carboxyl group. This peak becomes dominant in the spectra of AuNPs and AuNPs@RSV2, suggesting that the citrate and lysine carboxyls are found in their protonated form. Moreover, in the spectrum AuNPs the peaks at 1575 and 1380 cm^−1^ related to the deprotonated carboxylate group disappear completely.

The spectrum of AuNPs@RSV2 is dominated by the C=O stretching peak of protonated carboxyls, with minor contributions at 1650 and 1550 cm^−1^ that could be related to symmetrical and asymmetrical N-H bending of the ammonium cation of L-cys. The intense peak at 1450 cm^−1^ could indicate the presence of some deprotonated carboxylate anions. At lower wavenumbers, the spectrum shows peaks related to RSV (marked with * in Figure 4) at 1377, 1155, 950, 830 and 630 cm^−1^, yielding evidence of successful adsorption of the RSV molecule on the AuNPs surface.

#### 3.2.3. NEXAFS Characterization

**Figure 5:**
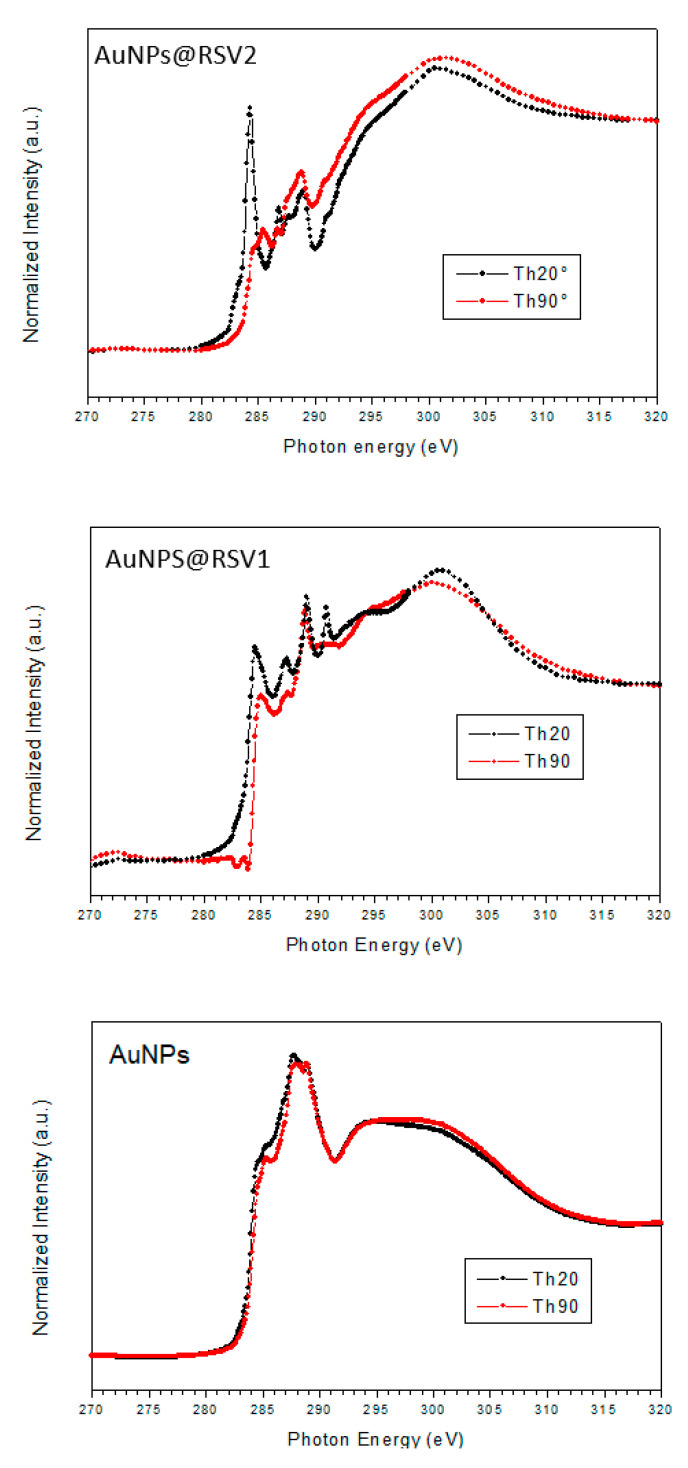
Near Edge X-ray Absorption Fine Structure (NEXAFS) C K edge NEXAFS spectra of samples AuNPs, AuNPs@RSV1 and AuNPs@RSV2 recorded at grazing (Th20) and normal (Th90) incidence.

C K edge NEXAFS spectra of AuNPs, shown in Figure 5, were recorded at normal and grazing incidence; however, no angle-dependent effects were detected.

The spectrum shows two intense peaks located at 287.7 and 288.8 eV, with a shoulder at 285.5 eV. According to literature, the 1s→π* transition related to the C=O bond in the carboxylate of cysteine is expected at 288.6 eV [61]; similar values are expected for carboxyl groups [62,63]. Moreover, L-cys is expected to show a 1s→σ* peak related to the C-S bond at about 287.3 eV. The broad bands located at about 294 and 300 eV are related to 1s→σ* transitions of C–H and C=O bonds, respectively [64].

In the spectra of AuNPs@RSV1 and AuNPs@RSV2 relevant changes are evidenced, clearly related to the conjugation of RSV on the nanoparticles surface. RSV (*trans*–3,5,4-trihydroxystilbene) is a complex molecule, consisting of a phenol and a 1–3 diphenol unity linked through a vinyl group, in a building block approach. According to the literature [64], the C k edge spectrum of the vinyl moiety should be dominated by the 1s→π* transition related to the C=C bond, located at 284.5 eV. The phenol moiety presents a more complex spectrum. For the benzene molecule, two distinct 1s→π* transitions are found in the C K edge spectrum (π*_1,2_ and π*_3_), located at about 285.4 and 289.2 eV, respectively (small shifts of ±0.2 eV can be detected depending on the aggregation state), the first peak being the most intense. The insertion of a heteroatom as substituent in the benzene ring causes the two mentioned peaks to split in two in systems like aniline or phenol, due to the higher Binding Energy of the carbon directly bonded to the heteroatom (C_1_) and of all the other carbons (C_2,3,4_). In the spectrum of phenol, four different 1s→π* transitions can be evidenced: π*1,2 (C_2,3,4_) at 285.4 eV, π*2 (C_1_) at 287.2 eV, π*3 (C_2,3,4_) at 288.8 eV, π*3 (C_1_) at 290.9 eV; for all the mentioned peaks small shifts of ± 0.2 eV can be detected depending on the aggregation state [64]. In a building block approach, we expect to detect a similar trend for the phenol unity of RSV and for the 1–3 diphenol unity, but with higher intensity for the transitions related to the C_1_ atom.

The main peaks evidenced in the 284–292 eV region of the C k edge spectrum of sample AuNPs@RSV1 are located at 284.8 eV, with a shoulder at 285.3 eV, and at 287.3, 288.9 and 290.7 eV respectively. The first peak results from the superimposition of the π*_1,2_ (C_2,3,4_) transition of phenol (285.4 eV), with the π* peak of vinyl in resveratrol, expected at 284.5 eV. The second peak is related to π*_2_ (C_1_), with possible contributions from the σ* peak related to the C-S bond of cysteine, located approximately at the same photon energy. The peak at 288.8 eV results from the π*_3_ (C_2,3,4_) peak of resveratrol, but also from substantial contributions of the π*_C=O_ transitions of cysteine and citrate that, according to FT-IR spectrum, is the main component of the surface of AuNPs@RSV1 nanoparticles. The last peak (290.7 eV) corresponds to the π*_3_ (C_1_) peak, with possible contributions from the K2p_3/2_ peak of potassium ions located on the surface, related to the presence of deprotonated citrate molecules, as evidenced by FT-IR spectroscopy.

All the mentioned peaks are clearly evident in the spectrum recorded at grazing incidence (Th20), but when the incidence angle is moved to normal (Th90) the intensity of all the peaks related to the π* transitions of resveratrol decreases, with the exception of the peak at 288.8 eV, whose intensity is mainly related to the π*_C=O_ transitions of cysteine and citrate. This result suggests the formation of ordered structures, possibly resulting from partial aggregation of RSV molecules. In the region above 292 eV, the two broad bands having σ* character located at 294 and 301 eV were already present in the spectrum of AuNPs, and assigned to 1s→σ* transitions of C–H and C=O bonds, respectively; phenol also shows two σ*_C-C_ bands in the same position. Slight angular effects are evidenced also on the σ* bands intensity.

In the 284–292 eV region of the C k edge spectrum of sample AuNPs@RSV2, peaks are located at 284.5 eV, with a shoulder at 285.3 eV, 286.7 eV, 288.8 eV, with a shoulder at 287.6 eV and a low intensity peak 290.6 eV. The features are similar to the ones evidenced in the spectrum of AuNPs@RSV1, without contributions from K2p signals. However, the angle dependency of the π* transitions, when the incidence angle is changed from grazing to normal, is striking, strongly suggesting the formation of ordered resveratrol aggregates on the AuNPs surface; this phenomenon appears more pronounced for AuNPs@RSV2 than for sample AuNPs@RSV1, due to the different preparation methodology, suggesting the formation of larger aggregates. This result confirms the previously discussed XPS and FT-IR data, which evidenced the formation of RSV aggregates on the AuNPs surface because of physisorption for sample AuNPs@RSV2.

### 3.3. AuNPs and AuNPs@RSV Biocompatibility

**Figure 6:**
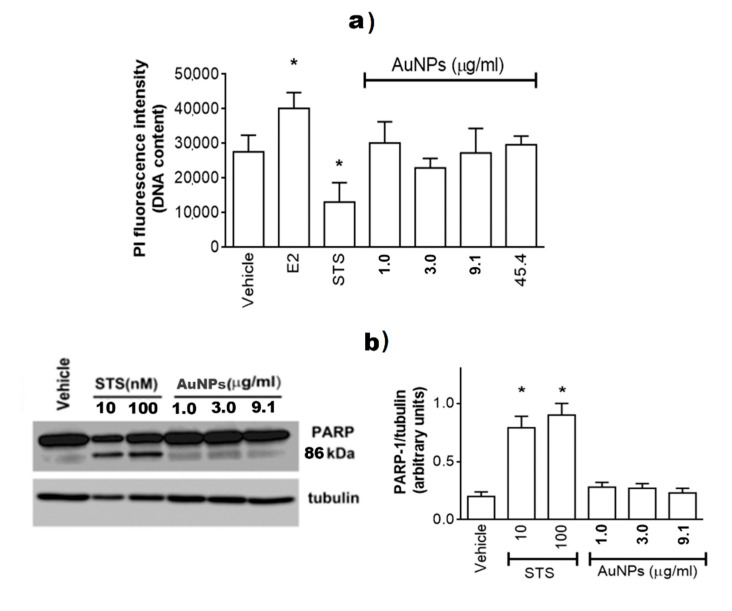
Analyses of cellular DNA content obtained from propidium iodine assay (PI). (**a**) MCF-7 cells were treated for 48 h with either vehicle (ethanol/DMEM, 1/10) or E2 (10 nM) or staurosporine (100 nM; STS) or with different AuNPs concentrations. Data are means ± SD of six different experiments. (*) *p* < 0.001 was calculated with Student’s *t* test versus vehicle. (**b**) Western blot (left) and densitometric analyses (right) of PARP-1 cleavage in MCF-7 cells. Cells were treated with staurosporine (10 or 100 nM, STS) or with different AuNPs concentrations. The amount of cleaved protein was normalized by comparison with α-tubulin levels. Data are the mean ± SD of five different experiments. (*) *p* < 0.001 was determined with Student *t*-test with respect to the vehicle.

Since the AuNPs–RSV2 system proved to be less controlled both dimensionally (DLS data) and structurally (by HR-XPS, FT-IR and NEXAFS data), the investigation on the cellular viability effectiveness of AuNPs was performed in breast cancer cells paralleled with the evaluation of anticancer effects of RSV and AuNPs@RSV1. The estrogen receptor α positive MCF-7, the growth of which is dependent on the hormone 17β-estradiol (E2), have been used as experimental model. As expected, E2 treatment increased the number of cells (Figure 6a), while the cell stimulation with the apoptosis inducer Staurosporin (STS) strongly reduced cell numbers. Notably, none of AuNPs concentration tested (i.e., 1.0–45.5 µg/mL) modified MCF-7 morphology (data not shown) or their vitality (Figure 6a). The AuNPs effect on cell vitality has been further confirmed evaluating the activation of poly (ADP-ribose) polymerase-1 (PARP-1) cleavage (Figure 6b). PARP-1, a DNA-binding enzyme involved in DNA damage processing, is one of several known cellular substrates of caspases. Cleavage of PARP-1 by caspases is a hallmark of the process of programmed cell death or apoptosis [65]. Only the apoptotic inducer (i.e., STS) promotes PARP-1 cleavage increasing, in a dose-dependent manner, the level of 89 kDa band, while concentrations of AuNPs significantly modify the level of cleaved PARP-1 (Figure 6b). These results sustain the incapability of AuNPs to trigger a stress-dependent signal transduction pathway that culminates in the activation of apoptotic cascade.

**Figure 7:**
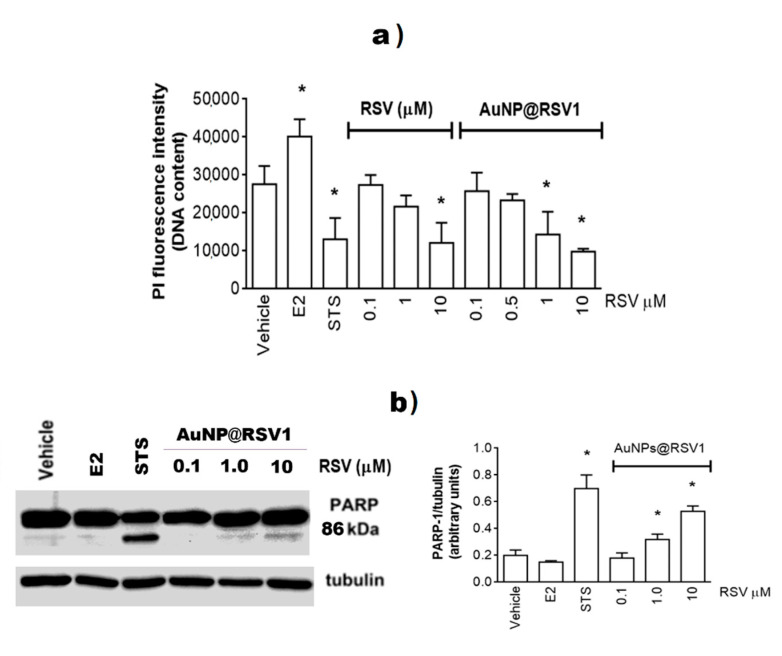
Analyses of cellular DNA content obtained from propidium iodine assay (PI). (**a**) MCF-7 cells were treated for 48 h with either vehicle (ethanol/DMEM, 1/10) or E2 (10 nM) or staurosporine (100 nM; STS) or with different RSV or concentrations. Data are means ± SD of six different experiments. *p* < 0.001 was calculated with ANOVA followed by Bonferroni test versus vehicle (a), versus RSV 0.1 and 1 µM (**b**), versus the same concentrations of AuNPS@RSV1 (c), versus AuNPS@RSV1 0.1 µM (d), and versus the same concentrations of RSV (e). (**b**) Western blot (left) and densitometric analyses (right) of PARP-1 cleavage in MCF-7 cells. Cells were treated with E2 (10 nM) or staurosporine (10 nM, STS) or with different AuNPs@RSV1 concentrations. The amount of cleaved protein was normalized by comparison with α-tubulin levels. Data are the mean ± SD of three different experiments. (*) *p* < 0.001 was determined with Student *t*-test with respect to the vehicle.

The efficacy of AuNPs@RSV1 as an anti-tumor agent was successively evaluated. The extensive anticancer effects ascribed to RSV seem to depend on several action mechanisms, the activation of which depends on RSV concentration. Indeed, RSV concentrations ranging from 50 to 300 µM could activate DNA repair mechanisms, modulate cell cycle progression, and influence the expression of apoptosis-related genes [31]. Most investigations have demonstrated that RSV concentrations (i.e., 10–20 µM) sensitize breast carcinoma cell lines to chemotherapeutic agents such as Paclitaxel by down-regulating surviving expression while increasing apoptosis [66]. RSV concentrations < 10 µM (i.e., 0.5–5.0 µM) have been reported to interfere with estrogen receptor α subtype activities devoted to E2-dependent breast cancer cell growth and survival [31]. Thus, to obtain more information on RSV action mechanisms, the AuNPs@RSV1 were produced by using specific amounts of RSV molecules (i.e., 0.5–10 µM) during AuNPs synthesis. At only 10 µM concentration, RSV reduces MCF-7 number (Figure 7a), whereas AuNPs@RSV1 decreases cell number already at 1.0 µM (Figure 7a). The concentration-dependent decrease in cell number induced by AuNPs@RSV1 is not a cytotoxic phenomenon being associated with the activation of the apoptotic cascade, as demonstrated by the PARP-1 cleavage. Indeed, a weak but significant increase of PARP-1 89 kDa band over the control is induced by stimulating MCF-7 cells with 1.0 µM AuNPs@RSV1, while a more robust level of cleaved PARP-1 is present after the treatment of MCF-7 cells with 10 µM AuNPs@RSV1 (Figure 7b). Notably, we previously showed that at a concentration of just 10 µM, RSV alone significantly increases PARP-1 cleavage [31]. The results provide compelling evidence that conjugated RSV maintains its anticancer effects even at very low concentrations, suggesting that the association with AuNPs could enhance RSV entry into the cells, as reported by other authors using gum Arabic stabilized RSV-encapsulated gold nanoparticles [42].

## 4. Conclusions

RSV is one of the most widely studied plant polyphenols for its potential, yet often controversial, health benefits. Nonetheless, most of the many RSV effects reported in in vitro studies have failed to be reproduced in vivo, mainly due to RSV pharmacokinetics. Indeed, in human beings, RSV is highly absorbed orally (~70%) yet has poor systemic bioavailability (~0.5%), being rapidly metabolized into RSV sulfate and glucuronide conjugates. Furthermore, gut microbiota and genetic background generate a wide range of inter-individual responses upon oral ingestion of RSV. In contrast, in vitro studies have described an array of mechanistic effects that generate controversy given the likely non-physiological concentrations used, as well as the omission of the contribution of RSV metabolites [34]. As for other drugs, the problem could be solved by exploiting highly hydrophilic AuNPs to deliver RSV.

Here, two approaches to produce AuNPs–RSV conjugate were studied in order to obtain a high load of RSV that can be transported and bioavailable. Above all, the interaction of AuNPs with RSV was investigated from a structural point of view, highlighting the key role of the surface of gold that combines RSV with non-covalent interactions. In addition, biological tests were carried out on AuNPs and the best conjugate to verify cytotoxicity and the maintenance of the biological activity of the loaded RSV. Large efforts are currently devoted to understanding the potential adverse effects of NPs on humans. Several key findings have been made recently. Among these, it has been observed that NPs may accumulate in the liver and kidney, triggering oxidative stress, inflammation, and indirect DNA damage in living systems. It has been proposed that once the metallic NPs are taken up in cells, they can release their cargo intracellularly, but the acidic environment of the lysosomes, in which NPs accumulate, triggers the release of relatively toxic ions (e.g., Ag^+^, Au^1+/3+^ ions) in the cell that are the true mediators responsible for the observed intracellular toxicity profiles [67]. However, the size, shape, and a stable coating of NPs may have a key role in determining the cellular damage. Indeed, while those effects were very pronounced in small (13 nm) sized gold nanospheres, citrate-coated 60 nm nanospheres were much less toxic and did not present most of the above reported organ and cellular alterations and always in a dose dependent manner [68]. In this paper, sphere-shaped 45.0 nm ± 12.0 AuNPs and AuNP@RSV1 were synthetized using citrate and L-cysteine as capping agents. None of tested NP concentration (up to 45.5 µg) induced cell death, suggesting that cellular alteration of our nanospheres may be low. These only preliminary in vitro data will need further validation.

Although the ability of AuNPs@RSV to maintain the anticancer effects of RSV against hepatoma and ERα negative cell lines growth, as well as against breast cancer invasion, has been reported [42,46,47], here our data demonstrated, for the first time, the ability of gold-conjugated RSV to reduce the ERα positive breast cancer cell number by inducing the activation of PARP-1 cleavage. Although more experiments, currently ongoing in our laboratory, are requested to better elucidate the action mechanisms of RSV released from AuNPs, our data provide compelling evidence that RSV on AuNPs enhances the bioavailability of this stilbene so that therapeutically active species can be optimally available in vivo at physiological concentrations, reducing the inter-individual differences and opening new avenues for applications in therapies against human diseases.

## Supplementary Materials

The following are available online at https://www.mdpi.com/2079-4991/10/10/1898/s1, Figure S1: Calibration curve of RSV in water; Figure S2: O1s, N1s, S2p and Au4f XPS spectra of AuNPs@RSV1; Table S1. XPS BE, FWHM, Atomic Percent values and Assignments.
