# Supplementary material for: Direct Conjugation of Resveratrol on Hydrophilic Gold Nanoparticles: Structural and Cytotoxic Studies for Biomedical Applications
Source: Nanomaterials (Basel). 2020 Sep 23;10(10):1898. doi: 10.3390/nano10101898 (PMC7598182; doi:10.3390/nano10101898)
Supplement: Supplementary file 1 [file nanomaterials-10-01898-s001.pdf]

nanomaterials

MDPI

Article

Direct Conjugation of Resveratrol on Hydrophilic Gold Nanoparticles: Structural and Cytotoxic Studies for Biomedical Applications

Iole Venditti [1,*], Giovanna Iucci [1], Ilaria Fratoddi [2], Manuela Cipolletti [1], Emiliano Montalesi [1], Maria Marino [1], Valeria Secchi [1] and Chiara Battocchio [1]

[1] Department of Sciences, Roma Tre University of Rome, 00146 Rome, Italy; giovanna.iucci@uniroma3.it (G.I.); manuela.cipolletti@uniroma3.it (M.C.); emiliano.montalesi@uniroma3.it (E.M.); maria.marino@uniroma3.it (M.M.); valeria.secchi@uniroma3.it (V.S.); chiara.battocchio@uniroma3.it (C.B.)

[2] Department of Chemistry, Sapienza University of Rome, 00185 Rome, Italy; ilaria.fratoddi@uniroma1.it

* Correspondence: iole.venditti@uniroma3.it; Tel.: +39-06-5733-3388

Received: 31 August 2020; Accepted: 18 September 2020; Published: 23 September 2020

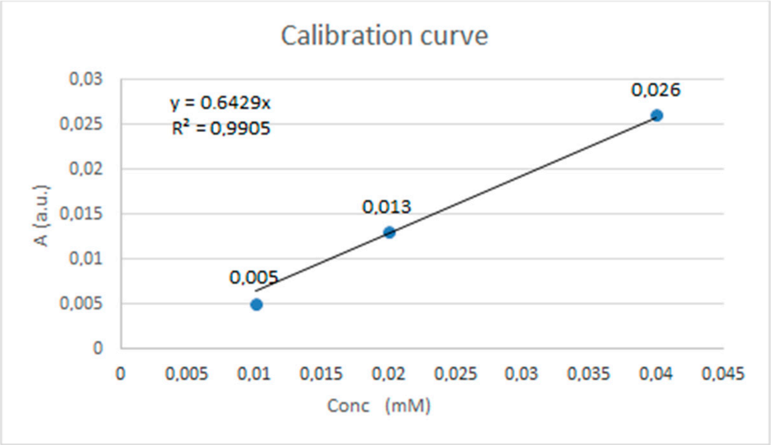

Figure S1. Calibration curve of RSV in water.

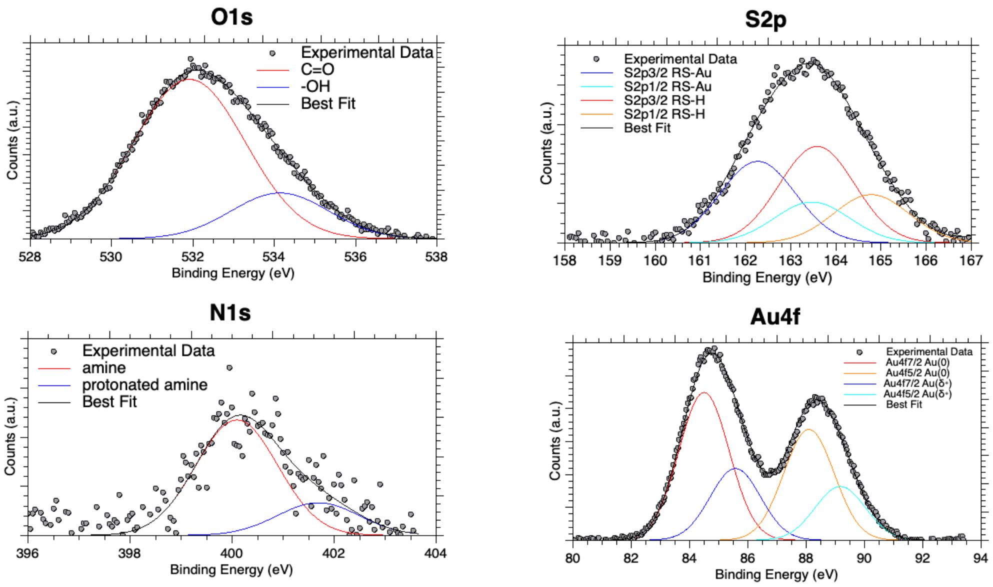

Figure S2. O1s, N1s, S2p and Au4f XPS spectra of AuNPs@RSV1.

Table S1. XPS BE, FWHM, Atomic Percent values and Assignments.

Sample	Signal	BE (eV)	FWHM (eV)	Atomic Percent (%) or Atomic Ratio (Oi/Oj)	Assignment
AuNPs	C1s	285.00	1.81	32.7	C–C
		286.82	1.81	28.2	C–N, C–S, C–O
		288.37	1.81	24.2	COOH
		290.07	1.81	10.1	COO–
		291.72	1.81	4.8	impurities
	$Au4f_{7/2}$	84.00	2.03	67.3	Au(0)
		85.07	2.03	32.7	$Au(^{+})$
	$S2p_{3/2}$	162.27	1.96	45.8	RS–Au
		163.59	1.96	54.2	RS–H
	N1s	400.13	2.50	71.7	RNH_2
		401.74	2.50	28.3	RNH_3^+
	O1s	531.93	2.85	3.0	O=C–
		533.34	2.85	1.0	–O–H
RSV	C1s	284.70	1.54	79.2	C–C
		286.38	1.54	11.4	C–OH
		289.00	1.54	9.4	COOH impurities
	O1s	529.66	1.62	-	TiO_2 (substrate)
		531.47	1.62	2.3	O=C–
		532.92	1.62	1.0	–O–H
AuNPs@RSV1	C1s	285.00	2.17	44.4	$C–C_{aliphatic+aromatic}$
		286.60	2.17	32.0	C–N, C–S, C–O
		288.45	2.17	18.5	COOH
		290.31	2.17	5.1	COO–
	$Au4f_{7/2}$	83.96	1.20	79.9	Au(0)
		84.97	1.20	23.1	$Au(^{+})$
	$S2p_{3/2}$	161.99	1.82	68.1	RS-Au
		164.11	1.82	31.9	RS-H
	N1s	400.10	1.89	80.0	RNH_2
		401.69	1.89	20.0	RNH_3^+
	O1s	529.47	3.03	-	TiO_2 (substrate)
		532.12	3.03	2.3	O=C–
		533.18	3.03	1.0	–O–H
AuNPs@RSV2	C1s	284.70	1.60	87.3	$C–C_{aliphatic+aromatic}$
		286.48	1.60	4.9	C–OH
		288.16	1.60	7.8	COOH
	$Au4f_{7/2}$	83.98	1.29	82.6	Au(0)
		85.00	1.29	17.4	$Au(^{+})$
	$S2p_{3/2}$	161.54	1.86	80.6	RS–Au
		163.79	1.86	19.4	RS–H
	N1s	399.18	2.51	100	RNH_2
	O1s	529.66	1.79	-	TiO_2 (substrate)
		531.20	1.79	4.2	O=C–
		532.87	1.79	1.0	–O–H
